# Temporal variability analysis reveals biases in electronic health records due to hospital process reengineering interventions over seven years

**DOI:** 10.1371/journal.pone.0220369

**Published:** 2019-08-07

**Authors:** Francisco Javier Pérez-Benito, Carlos Sáez, J. Alberto Conejero, Salvador Tortajada, Bernardo Valdivieso, Juan M. García-Gómez

**Affiliations:** 1 Biomedical Data Science Lab, Instituto Universitario de Tecnologías de Información y Comunicaciones Avanzadas (ITACA), Univeritat Politécnica de València, València, Spain; 2 Instituto Universitario de Matemática Pura y Aplicada, Universitat Politécnica de València, València, Spain; 3 Unidad conjunta de investigación en reingeniería de procesos socio-sanitarios, Instituto de Investigación Sanitaria La Fe, Hospital Universitario La Fe, València, Spain; 4 Red de Investigación en Servicios de Salud en Enfermedades Crónicas (REDISSEC), València, Spain; Medical University Graz, AUSTRIA

## Abstract

**Objective:**

To evaluate the effects of Process-Reengineering interventions on the Electronic Health Records (EHR) of a hospital over 7 years.

**Materials and methods:**

Temporal Variability Assessment (TVA) based on probabilistic data quality assessment was applied to the historic monthly-batched admission data of Hospital La Fe Valencia, Spain from 2010 to 2016. Routine healthcare data with a complete EHR was expanded by processed variables such as the Charlson Comorbidity Index.

**Results:**

Four Process-Reengineering interventions were detected by quantifiable effects on the EHR: (1) the hospital relocation in 2011 involved progressive reduction of admissions during the next four months, (2) the hospital services re-configuration incremented the number of inter-services transfers, (3) the care-services re-distribution led to transfers between facilities (4) the assignment to the hospital of a new area with 80,000 patients in 2015 inspired the discharge to home for follow up and the update of the pre-surgery planned admissions protocol that produced a significant decrease of the patient length of stay.

**Discussion:**

TVA provides an indicator of the effect of process re-engineering interventions on healthcare practice. Evaluating the effect of facilities’ relocation and increment of citizens (findings 1, 3–4), the impact of strategies (findings 2–3), and gradual changes in protocols (finding 4) may help on the hospital management by optimizing interventions based on their effect on EHRs or on data reuse.

**Conclusions:**

The effects on hospitals EHR due to process re-engineering interventions can be evaluated using the TVA methodology. Being aware of conditioned variations in EHR is of the utmost importance for the reliable reuse of routine hospitalization data.

## Background and significance

### Introduction

A business process is defined as a structured set of activities performed in any organization for the description of the logical order and dependence of the processes carried out [1]. In healthcare organizations, business Process Reengineering means improving organizational performance by process or information system redesign, covering the needs of healthcare institutions [2–7]. Business process redesign has been applied in many healthcare systems such as pharmacies [8] and emergency departments [9] to increase their efficiency since they are now under pressure all over the world [10]. The authors of the review [11] showed that many of the studies that address the promotion of business process reengineering in the health sector are related to the reduction in the length of hospitalization or the help with organizational change and how this promotion may drive the development of similar actions, that seek to improve the quality of the services offered, in other organizations.

The data used to evaluate the population’s health underlies the effects of the decision-making processes that rely upon these data [12]. When assessing data quality in health systems, one of the most commonly examined dimensions is timeliness [12,13], which are considered to be an extrinsic data quality concept influencing fitness-to-use features [14,15].

Our aim was to make a descriptive and retrospective analysis about the process reengineering interventions influence on EHR, and to analyze how these interventions might have influenced hospital activities focusing on the potential technical knowledge which may be extracted from data. The TVA methodology was applied to a database that collects information on admissions to the *Hospital Universitario y Politécnico La Fe* (HFE) in Valencia between January 2010 and December 2016.

As will be discussed in Section *Discussion*, many works in recent literature are usually centered in one process and measures how well the intervention is working. Meanwhile, this study count on the main objective of applying a well-documented methodology for the evaluation of temporal variability [16–20] based on Information Geometry, not only to measure the influence of one process reengineering intervention but also to automatically detect interventions through data distributions.

## Materials and methods

### Ethics

This study did not involve any risk or changes to the healthcare services to patients and did not alter their regular intervention and treatment. Only authorized persons obtained data from electronic health records. They maintained the privacy and security of patients’ personal information by encoding their identity with dissociated non-traceable codes. This research was carried out in accordance with the International Guideline for Ethical Review of Epidemiological Studies [21] and the Biomedical Research Ethics Committee of the HFE [22], which approved the study protocol on October 10^th^, 2017 under the name “ANÁLISIS DE LA CALIDAD Y VARIABILIDAD DE DATOS MÉDICOS” (Registration Number 2017/0482).

### Materials

The study considered the hospitalization data repository of the HFE, in Valencia, Spain, including 108,347 admissions from 2010 to 2016. The HFE coordinates all public healthcare services provided by *La Fe Valencian Health Department*, from primary to tertiary care, covering 300,000 inhabitants directly and adding up to 515,000 persons from the catchment area. The HFE is the biggest reference hospital in the *Comunitat Valenciana* and the fifth largest in Spain. The HFE department is composed by the HFE (with 1,000 beds approx.), the health center of Campanar, located in the old facilities of the HFE, the specialty center Ricardo Trénor Palavicino and 20 primary health centers. The health department is met by a team of more than 7,000 people, that includes more than 1,100 doctors, 400 Internal medicine residents, 3,800 positions of different nursing areas and 1,500 people for management and general services.

The repository includes healthcare information on each hospital admission of the overall population during the aforementioned period. After gathering the data, we excluded the episodes of isolated patients, i.e. those who did not belong to the HFE department (for example tourists who are visiting the city), because of the possibility of missing significant information for the study, such as 30-day unplanned readmission or the diagnosis of chronic diseases prior to the date of admission.

Before conducting the TVA, a preprocess was carried out on some administrative and clinical variables. The original dataset was completed with some aggregate and processed variables, including the age of the patient which was computed as the difference between the admission date and their birth date, the Charlson comorbidity index score[23] that was calculated using updated weights from Schneeweiss, [24] and the ICD-9-CM coding, as proposed by Quan, [25]. This score was calculated by adding 1 point for the patient’s history of acute myocardial infarction, peripheral vascular disease, cerebrovascular disease and diabetes without complications; 2 points for congestive heart failure, chronic obstructive pulmonary disease, mild liver disease, diabetes with complications and malignancy; 3 points for dementia and renal disease; 4 points for moderate-to-severe liver disease and HIV infection; and 6 points for metastatic cancer. This score was calculated using another repository that included the ICD-9-CM code for each diagnosis of chronic disease recorded in the HFE.

The list of variables considered is shown in Table 1. Extra information on the materials can be found in the S1 Appendix.

**Table 1:** List of variables contained in the study case.

Variable	Description	Type (values/format)
Sex	Sex of the person	Discrete (Male, Female)
Age	Age in years at the time of the admission	Numerical Integer
AdmissionServiceCode	Code of the service of hospitalization	Discrete 4-length alphanumeric code
RealServiceCode	Code of the service related to the episode	Discrete 4-length alphanumeric code
DischargeServiceCode	Code of the service which discharged the patient	Discrete 4-length alphanumeric code
AdmissionTurn[1,2,3]	Admission shift	Discrete
AdmissionReason	Reason for hospital admission	Discrete (See S1 Appendix)
DischargeDate	Date of patient discharge	Date (yyyy/mm/dd)
DischargeTurn[1,2,3]	Discharge shift	Discrete
DischargeReason	Reason for patient discharge	Discrete (See S1 Appendix)
DischargeDestination	Destination after patient discharge	Discrete (See S1 Appendix)
DischargeBefore12	Discharge before 12:00 noon	Discrete (Yes, No)
Exitus	Death of the patient during hospitalization	Discrete (Yes, No)
Exitus 48	Death of the patient within two days after hospitalization	Discrete (Yes, No)
Hospital Transfer	Existence of hospital transfer	Discrete (See S1 Appendix)
LengthOfStay	Length of stay of hospitalization episode. It is measured by the number of nights that the patient was admitted.	Numerical Integer
Intervention	Surgical Intervention	Discrete (Yes, No)
PreoperatoryStay	Length of stay before the intervention	Numerical Integer
Readmission30	Was the patient readmitted during the 30 days after discharge?	Discrete (Yes, No)
CharlsonIndex	Charlson comorbidity index for hospitalization	Numerical Integer

The shift in which the patient is admitted and discharged is coded as 1 for the morning (from 8:00 am to 3:59 pm), 2 for the evening (from 4:00 pm to 11:59 pm) and 3 for the night (from 0:00 am to 7:59 am)

### Methods

#### Theoretical background

A systematic TVA methodology based on probabilistic data quality control was applied [19,20,26]. This methodology uses methods based on Information Geometry [27,28] which provide a way for the comparison of dissimilarities between probability distributions of different temporal data batches.

It firstly consists of modeling Probability Density Functions (PDF's) -in our case, it was made by the use of Kernel Density Estimation [29]-. The Jensen-Shannon distance (JSD), which is a symmetrized and smoothed version of the Kullback-Leibler divergence [30,31], provides a way to measure how different the non-parametric PDF's are.

The space in which each point represents one PDF and the distance between two points is that defined by the aforementioned distance, forms a simplex and is known as statistical manifold and possesses good mathematical properties [27].

**Fig 1:**
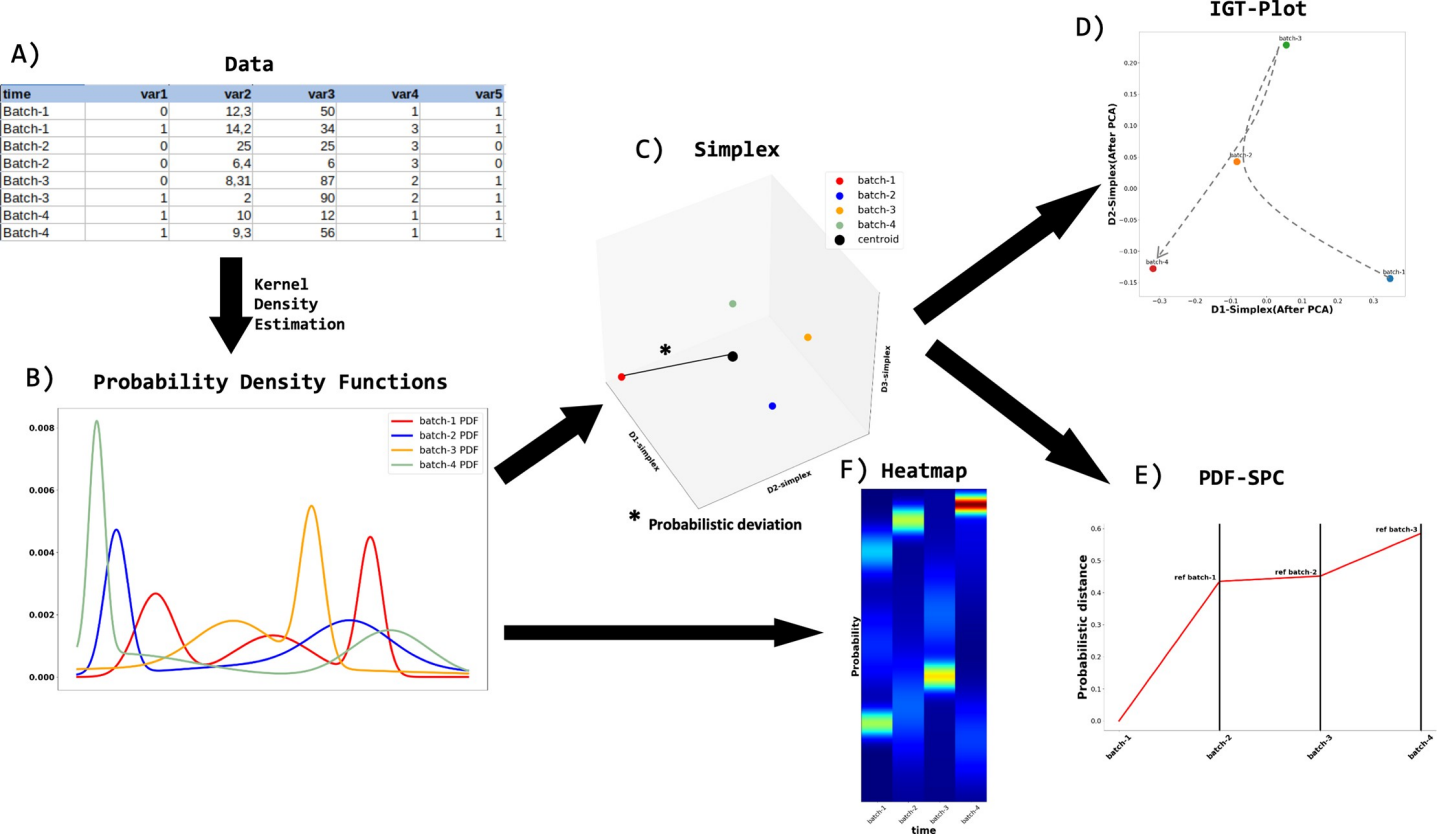
Technical diagram. The TVA methodology is based on information geometry. A short artificial experiment taking 4 temporal batches (only 4 batches were taken to ensure that the simplex could be represented in three dimensions) was drawn with the purpose of clarifying the concept. A) represents the generated artificial database in which binary, quantitative and continuous variables cope with. B) is the PDF representation. C) shows the simplex in which each point represents the PDF of one batch and the bigger black point represent the centroid of the simplex (the distance from each batch to the centroid serves as a dispersion measure). D) is the IGT-plot, in studies with more batches is one way to graphically represent the variability among the batches and to apply clustering methods to automatically detect temporal patterns, it must be noted that the color changes from previous representations to simulate the seasonal color mapping. E) shows the PDF-SPC, since the database was designed to present high variability, all the batches are “out-of-control”. Finally, F) presents the heatmap of the concatenated batches distributions which allows monitoring temporal pattern changes.

This function representation allows us, for example, to compute the centroid of the PDF's and to apply projection methods, such as Principal Component analysis [32] or Multidimensional Scaling [33,34]. These artifacts, as can be seen in Fig 1 where a short artificial experiment was driven to yield a simple proof of concept, give us the possibility of quantifying the dispersion and making space representations as a graphical way to detect variability. The exploratory methods provided by the methodology are:

- **Information Geometry Temporal (IGT) plot:** This presents a visualization of the temporal evolution of data. Temporal batches are laid out as a 2D plot while conserving the dissimilarities among their distributions. The IGT plot helps to reveal temporal trends in the data (as a continuous flow of points), abrupt changes (as an abrupt break in the flow of points), recurrent changes (as a recursive flow through specific areas), conceptually related time periods (as grouped points) and punctual anomalies (as isolated outlying points). Temporal batches are also labeled to show their date. They give seasonal information by means of colored labels (warm colors for summer and cool colors for winter) and are supported by a smoothed timeline path joining them [26]. The Density-based spatial clustering of applications with noise (DBScan) [35], was applied to the IGT plot using the median of the JSDs as grouping coefficient in order to automatically find temporal groups.
- **Probabilistic Statistical Process Control (PDF-SPC) algorithm:** The purpose of PDF-SPC is to monitor the degree of change in data variability distributions throughout consecutive temporal batches (in our case months), to a moving reference distribution -initially the first batch. According to the magnitude of the current change, measured by the JSD with respect to the reference distribution, the degree of change of the repository is classified into three states: in-control (distributions are stable), warning (distributions are changing), and out-of-control (recent distributions are significantly dissimilar to the reference, leading to an unstable state and yielding a change in the reference distribution). When an out-of-control state is reached, a significant change is confirmed and the reference distribution is set to the current one for subsequent comparisons. The warning and out-of-control states are represented as broken and continuous vertical lines, respectively.
- **Temporal Heat Maps:** Temporal Heat maps show the absolute or relative frequencies over time. The Temporal Heat map of a variable is a 2D plot in which the X-axis represents the time, the Y-axis represents a possible data value or range of values of the variable, and the color of the pixel at a given (X, Y) position indicates the frequency at which value Y was observed on date X. These heat maps facilitate a rapid broad visualization of the evolution over time of the values of the given variable.

The TVA methodology consists of using these methods iteratively. In a top-down approach, we start by analyzing the temporal variability of the complete monthly-batched data set. We then drill down to the specific variables or groups of variables which best explain the variability detected, according to the results of the analysis and prior knowledge of the repository.

#### Working methodology

This study was carried out by a multidisciplinary team of professionals from various fields: the technical background was provided by a computer scientist, a statistician, a mathematician, and specialist physicians whose expertise is the PR and the management of the hospital.

The study protocol was divided into two stages: in the first changes were detected and in the second one, they were analyzed and their causes were searched.

**Fig 2:**
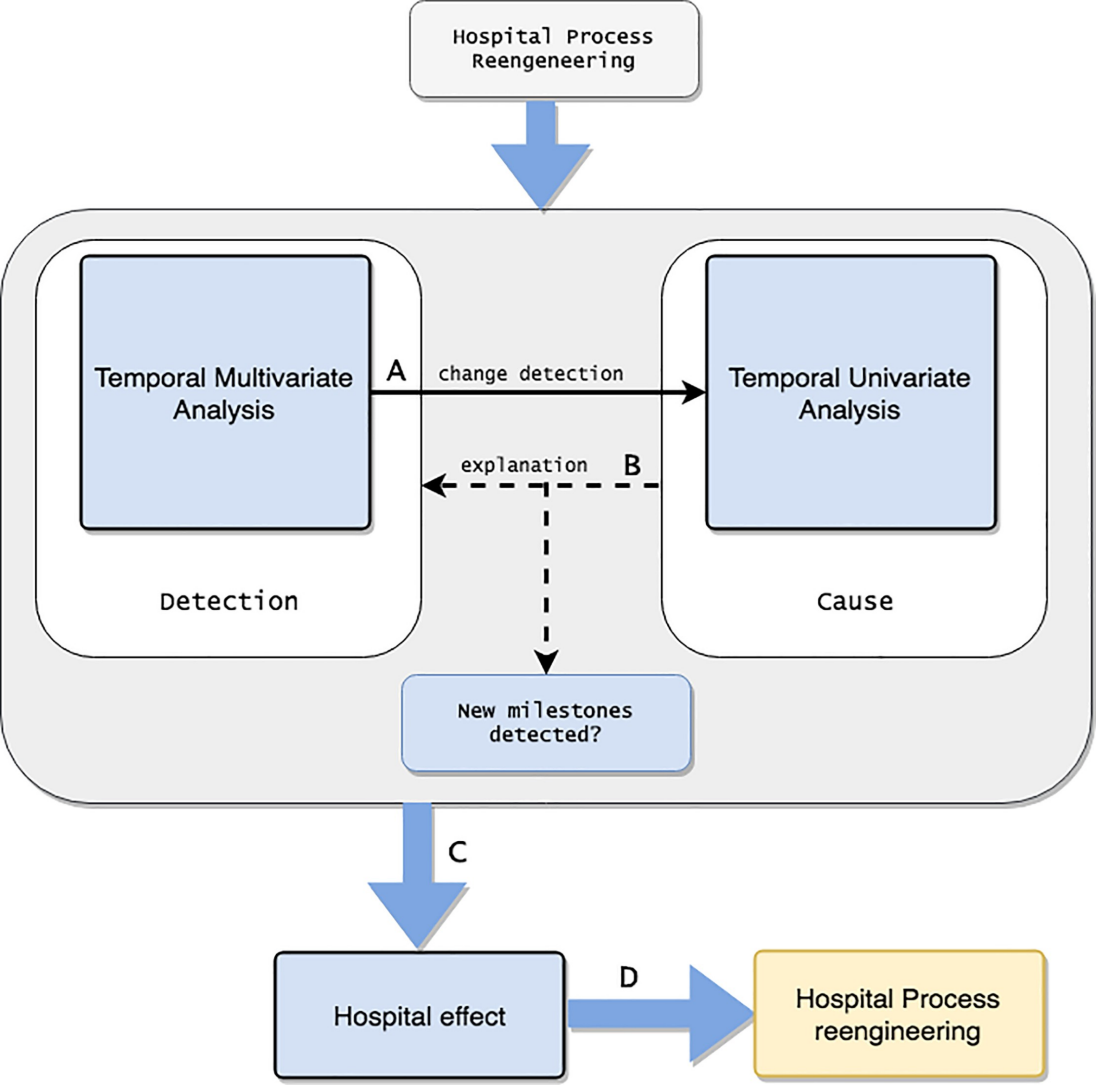
Work-flow diagram. Multivariate analysis is able to discover changes driven by the global probabilistic variability A). The obtained findings drive us to make the univariate analysis with the purpose of explaining the aforementioned changes B). It is worth mentioning that this step detects smoothed changes which had been covered by more abrupt global differences. Step C) is the evaluation of the interventions which provoked the data change and their implications. Finally, this evaluation could serve as the starting point for the implementation of PR D).

An overview of the study protocol is shown in Fig 2, in which the iterative protocol used for the detection of process reengineering interventions is described.

Following the previously described TVA methodology, we start by considering the whole multivariate dataset grouped by monthly batches under the assumption that PR interventions may imply an impact on EHR. This is intended to detect different data behavior patterns (see A) in Fig 2). Secondly, the same methodology was applied to the detected temporal data changes with a univariate approach to identify the variable, or set of variables, that could have influenced the observed global change. Subsequent automatic iterations for each variable may identify more univariate pattern changes which could have been smoothed due to multivariate batches with a greater global impact. These iterations can also detect interactions in the variables produced by changes in one variable (see B) in Fig 2).

## Results

We provide the description of how the proposed methodology was able to detect the effects, through data analysis, of the process reengineering interventions which will be shown in Section *Process reengineering interventions*. The list of findings mapped to the PR interventions carried out in the hospital in that period is shown in Table 2 in which the numerical evidence was added.

**Table 2:** Findings.

Finding	Intervention	Evidence
F1	I1—Hospital relocation	➢ IGT-plot and its DBScan clustering show differences between 2010 and the rest of the years of the study (Fig 3) (Multivariate JSD(10D, 11Jan) = 0.74, 2010 belongs to the green cluster and the rest of years belong to the blue cluster). ➢ Heat map of the PCA dimension reduction of the multivariate analysis offers an absolutely different pattern during the months of 2010 (Fig 3). ➢ PDF-SPC of AdmissionService, DischargeService, and RealService show the abrupt change detected at the end of 2010 (Fig 4). (AdmissionService univariate JSD(10Jan, 11Jan) = 0.26, DischargeService univariate, JSD(10Jan, 11Jan) = 0.26 and RealService univariate JSD(10Jan, 11Jan) = 0.25)
F2	I2—Services reconfiguration	➢ The heat map shows a trend of refinement of the red central band in the closest months to February 2011 (Fig 3). ➢ February 2011 is detected as an outlier by the IGT-plot and its DBScan clustering (Fig 3) (Multivariate JSD(11Jan, 11F) = 0.41 and Multivariate JSD(11F, 11m) = 0.73, besides DBScan did not assign to any cluster). ➢ PDF-SPC of AdmissionService, DischargeService, and RealService show the abrupt change detected at the beginning of 2011 (Fig 4). (AdmissionService univariate JSD(11Jan, 11m) = 0.33, DischargeService univariate, JSD(11Jan, 11m) = 0.29 and RealService univariate JSD(11Jan, 11m) = 0.29)
F3 ^a^	I3—Care services reconfiguration	➢ Heat map marks a different pattern of three months in mid-2013 (Fig 3). ➢ PDF-SPC of AdmissionService, DischargeService and RealService show the abrupt change detected in mid-2013 (Fig 4). (AdmissionService univariate JSD(13M, 11m) = 0.34, DischargeService univariate, JSD(13M, 11m) = 0.34 and RealService univariate JSD(13M, 11m) = 0.34) ➢ PDF-SPC, IGT-Plot and its DBScan clustering display an abrupt change in mid-2013 (Fig 5). (DischargeDestination univariate JSD(11a, 13m) = 0.29, two clusters well-defined of months prior to March 2013 and the rest).
F4	I4—Inclusion of 80,000 patients. The update of the pre-surgery admission protocol	➢ DBScan applied to IGT-plot warns of the existence of a month–January 2014- with an atypical behavior (Fig 3) (Unassigned month). ➢ This atypical month is also detected by the Heat map (Fig 3). ➢ PDF-SPC, IGT-Plot and its DBScan clustering show that a change occurred in early 2014 (Fig 6). (DischargeDestination univariate JSD(11m, 14F) = 0.22, two clusters well-defined of months prior to February 2014 and the rest) ➢ January 2015 is also detected as an atypical month by IGT-plot and its DBScan clustering (Fig 3). (Unassigned month) ➢ The Heat map analysis reveals that the number of hospitalizations increased from this month. It can be seen thanks to the width of the red band and is supported by the increment of admissions detected in 2015 (Table 2).
F5 ^a^	I3	➢ PDF-SPC, IGT-Plot and its DBScan clustering show an abrupt change in mid-2016 (Fig 5) (DischargeDestination univariate JSD(14F, 16M) = 0.24).

These findings were directly observed from data after the application of the methodology described in Section *Methods*.

^a^ The finding F5 was a direct cause of the intervention carried out and detected by the finding F3 (it will be discussed after).

### Findings

We define *finding* as a data-driven change. An observed finding in the data leads to the search for its cause and the assessment of these causes by hospital managers may identify future lines of work in terms of PR.

**Fig 3:**
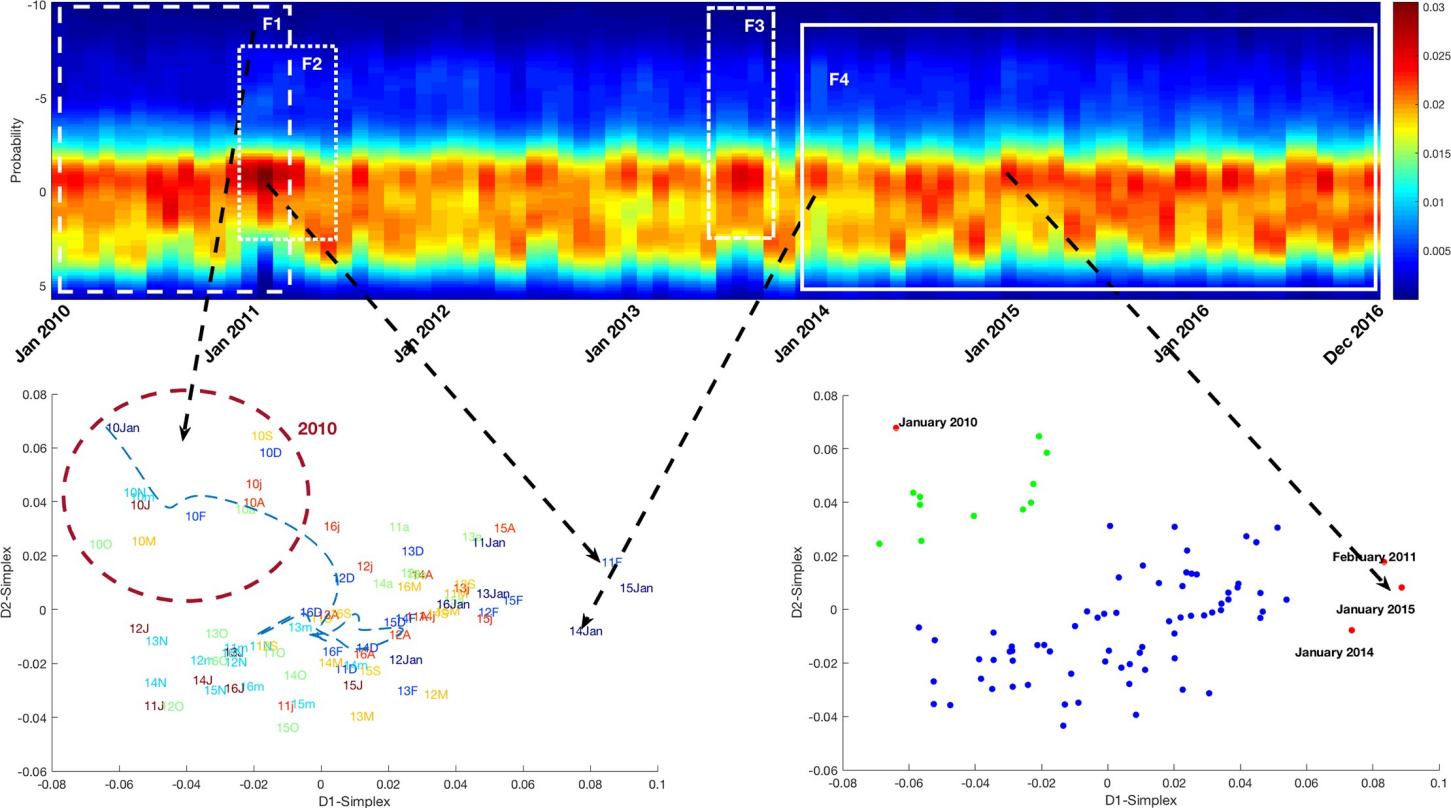
Multivariate analysis of hospitalizations in HFE. Four findings were detected. The top figure presents the heat map of the temporal multivariate data distribution, down left figure shows the IGT plot where the whole set of variables were considered and finally the DBScan clustering of IGT plot is exhibited in right down figure. F1 is correlated with the difference between 2010 and the rest of the years; F2 is aligned to data changes in early 2011; F3 stands for three months in mid 2013 with atypical patterns; F4 refers to January 2014, which is quite different from other months and introduce the beginning of an atypical pattern; the outlier detected in January 2015 is the precursor of the increase of frequency observed in the subsequent months. By analyzing the IGT-Plot and its clustering, we discovered that the heat map of the one dimensional PCA presented temporal color patterns.

As can be seen in Fig 3, relevant findings were detected by multivariate analysis. It is worth mentioning that seasonality in the data may be identified by the sinusoidal shape of the color map border.

In Fig 3 it can also be seen that the HFE probably suffered at least one important change in late 2010 (F1) and early 2011 (F2) that caused an abrupt change in all the monthly variable distributions, another significant event can be detected at mid-2013 (F3) where a density condensation may be observed on the top of the maximum-frequency band. Finally, at the beginning of 2014 (F4), an atypical month is detected, this month is followed by temporal patterns that had not been observed before and that led to an increment of the frequency from early 2015, 2015 January is detected as an outlier in the right down picture in Fig 3.

The univariate methodology was used in pursuit of an explanation for these changes. The changes can be explained by almost all the variables.

**Fig 4:**
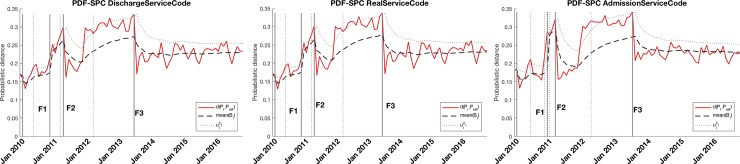
PDF-SPC of the three variables related to services configuration (Admission, Real and Discharge Service). Findings F1, F2, and F3 are detected in the three variables by out-of-control states.

Fig 4 shows that the variables which store the admission, real and discharge service of each hospital episode explain F1 and F2. The configuration of the hospital services may also explain Finding F3. The PDF-SPC’s of the services configuration is shown in this figure.

After removing the cases prior to March 2011, the same methodology was applied in order to avoid the non-detection of findings by the smoothing, which could have caused the abrupt changes prior to this date. As already mentioned, the changes previous to March 2011 had an impact on the whole set of variables.

**Fig 5:**
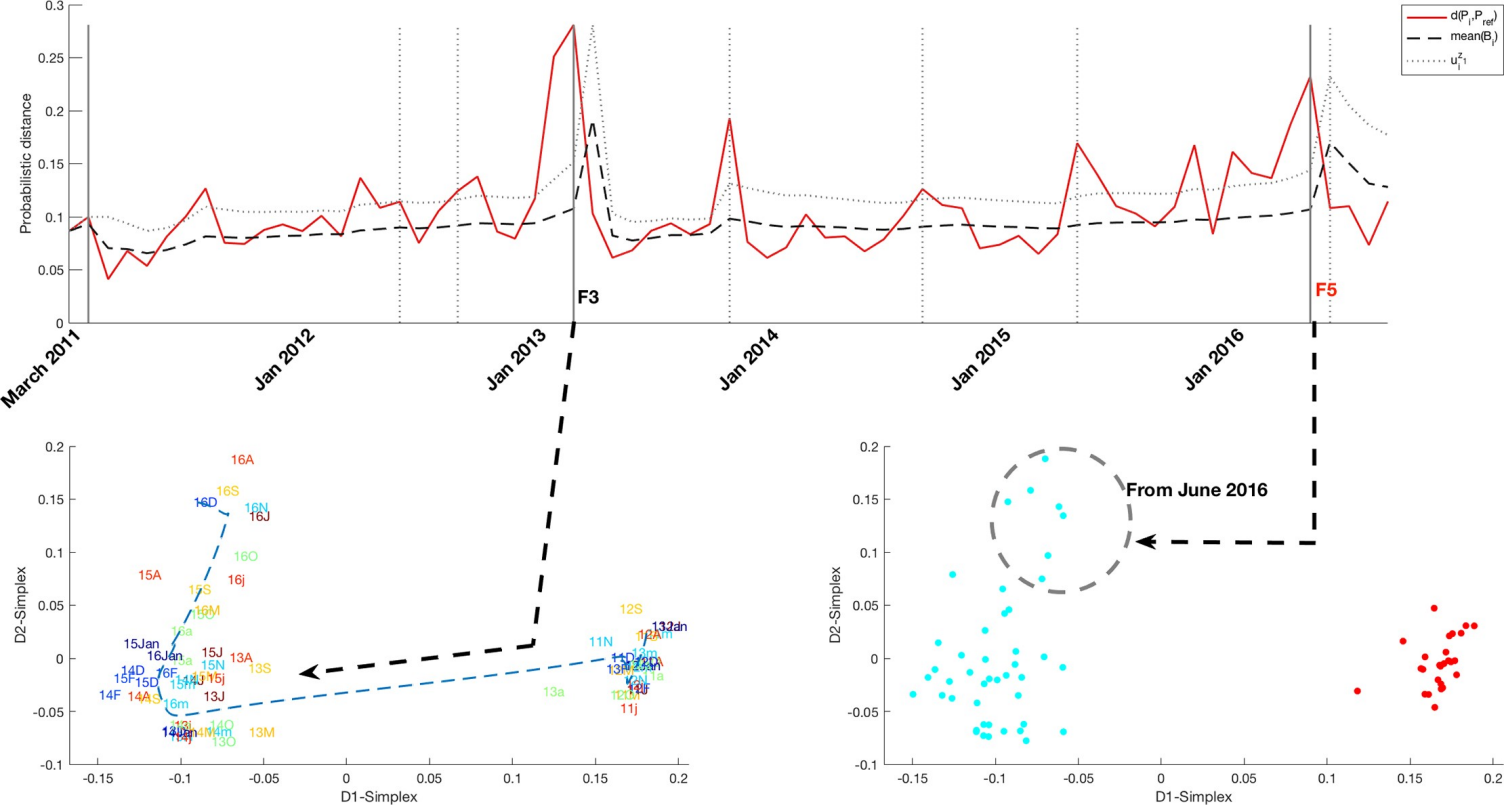
**PDF-SPC (top figure), IGT plot (left down figure) and its clustering by DBScan (right down figure) of the variable which records the method of patient follow-up after discharge (DischargeDestination).**The analysis of this variable shows new evidence for Finding F3 as well as a new Finding F5 (a new milestone is detected as mentioned in Fig 1) which probably was not detected by the multivariate analysis due to the higher hospitalizations from 2015 January.

Fig 5 shows the PDF-SPC, IGT Plot and its DBscan clustering for the variable DischargeDestination, showing the change of the discharge policy introduced between early-mid 2013 and mid-2016 which will be discussed in the next Section. This change is probably related to Finding F3 and will be referred to as Finding F5 (F5 is a new milestone -understanding milestone as different data distribution pattern- detected by applying the univariate methodology. The emergence of new milestones can be seen in Fig 2). Two temporal clusters were found.

**Fig 6:**
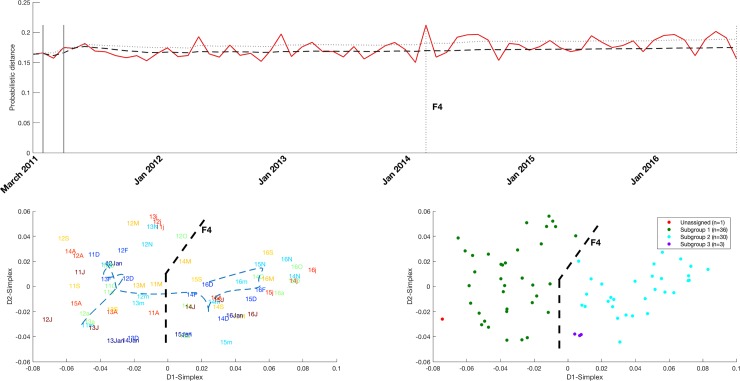
**PDF-SPC (top figure), IGT plot (left down figure) and its clustering by DBScan (right down figure) of the variable which measures the number of hospitalization days (LengthOfStay).** A change in the length of stay occurred in early 2014, related to Finding F4 was discovered in the multivariate analysis.

The exploratory PDF-SPC visualizations, IGT Plot and its DBscan clustering for the LengthOfStay variable are shown in Fig 6, where a variation in the patients’ average length of stay in early 2014 can be seen correlated with Finding F4. The histograms of this variable show an increase of 1-day stays with respect to 2 and 3-day stays (see S7 Fig).

The number of annual hospital admissions is shown in Table 3. It can be seen that the number of patients increased significantly from 2015 and this could have caused the change detected in the multivariate analysis (see Fig 3).

**Table 3:** Hospital admissions inflow.

Year	Number of admissions
2010	14,706
2011	12,969
2012	14,212
2013	14,459
2014	14,295
2015	18,063
2016	19,643
Total	108,347

Number of patients admitted per year

## Discussion

Healthcare organizations are constantly forced to increase the quality of care while maintaining an optimum use of resources [2,5]. Therefore, managerial decisions, which are routinely taken in a business environment, are constantly influencing data distributions. These decisions may imply temporal variability inherent to the data. In this field, the impact may not only be on the hospital management, but also on the regular population health and on the perception of its quality [36–39].

There exist some approaches to carry out the assessment of process reengineering interventions on literature. The authors of [40] propose a methodology based on process-mining to measure the organizational changes in the stroke emergency process. The assessment was performed by the use of PALIA [41]. One of the most powerful tools for process-mining is PROM [42] which covers a wide range of process-mining algorithms such as α-algorithm [43], genetic process-mining [44] or Heuristic Miner [45].

The present study searches something similar but is based on a consistent methodology driven by the variations of PDF applied to a health service dataset with the purpose of studying the effect of PR interventions on data. This methodology is used to monitor data distributions through time, becoming a way for “real-time” detection of the impact of management decisions and process reengineering interventions on hospital activities as well as finding undesired factors or effects [46]. We think that the principal Impact of our methodology is its global applicability when compared to the aforementioned approaches. These approaches are usually centered on one process and measures how well the intervention is working. Meanwhile, the proposed methodology provides the capability of detecting the own interventions by the multivariate iteration and its influence (not only direct but also indirect) in other related processes by the univariate iteration.

Besides, another contribution is that the detection of data distribution changes can lead to the improvement of future decisions and research work, for instance, a 30-day readmission model or the development of longitudinal studies could be better built from the prior knowledge of the findings of our study.

Although some of the milestones that have been detected are not the result of process re-engineering, but rather are specific daily situations that influence the operation of the hospital. These milestones have been taught because we believe that these situations could motivate one or more interventions in terms of process re-engineering. We also remark that HFE experts in PR analyze the impact on hospital management as well as on the regular patient population’s health by exploring the reasons and the effects on hospital activities of decisions already taken (see C) in Fig 2). Although the following is outside the scope of this work, the results of this analysis may help to identify indicators which could be the input for further PR decisions (see D) in Fig 2).

A list of the process reengineering interventions, contributions, limitations and lines of future work are given below.

### Process reengineering interventions

The process reengineering interventions carried out by the hospital managers and their motivation are presented in chronological order with the purpose of correctly interpreting the findings, shown in Section *Results*, that the exploratory method applied was able to detect.

#### Hospital relocation (I1)

The HFE relocated to new facilities between December 2010 and February 2011, which involved a progressive reduction of admissions that lasted while the intervention finished, the time when hospital activity recovered. The relocation protocol was the following:

- The Outpatient Department was relocated on November 2010. The first allergy, dermatology, internal medicine, and infectious disease consultations took place on November 29^th^ (finding F1).

The remaining areas were progressively moved from lower to higher logistical complexity. Finishing with the transfer of the most delicate areas as follow:

- Maternity and Child Health was transferred on February 13^th^, 2011, moving 81 children and premature babies and 11 pregnant women and recently delivered mothers (finding F2).
- The adult hospitalizations area was relocated on February 20^th^, 2011, with 158 adults (finding F2).

Consequently, after December the admission-patient typology became urgent profiles (see S1 and S2 Figs). The admission of patients with a higher age and comorbidity index was caused by the relocation since this type of patient frequently has a serious illness and requires more urgent resources. The number of interventions decreased, as allowed for in the managers’ planning (see S2 Fig). After opening the new facilities, more hospital transfers were (see S3 Fig) needed and the information system was changed. The admission planning taken by the hospital management for the relocation was quite similar to the interventions adopted during the summer months, in order to allow for staff holidays, which can also be detected by the seasonality in the data.

#### Services re-configuration (I2)

At the beginning of 2011 (Finding F2) and closely related to the previous point, the services were restructured (see S4 Fig) when the old facilities composed of four hospital centers were combined into one. The services were reorganized into clinical management areas and a committee for the approval or rejection of changes in service configuration was created.

#### Care services distribution (I3)

Despite the abovementioned relocation, some of the patients were still treated in the old facilities, as in the case of chronic patients, since it was decided to send them to the old facilities for patient follow-up at the beginning of 2013. This intervention involved a new service re-structuring and a higher percentage of patients were sent to their general practitioner in detriment of those discharged home for follow-up (finding F3, see Table 2). This situation was temporary due to the closure of the previous chronic unit in mid-2016 (finding F5, see Table 2), which meant more patients were monitored at home. A higher quantity of resources, therefore, had to be allocated to this end (see S5 Fig). Fig 3 served to detect this intervention and allowed us to suspect that a new cluster would probably have appeared from mid-2016 if the following months had been added to the dataset.

#### Changes in the pre-surgery admission protocol due to the inclusion of patients from another hospital (I4)

Another important intervention adopted by the hospital management in 2015 was due to prior knowledge of the assignment to the HFE of approximately 80,000 patients (now the hospital covers around 280,000 inhabitants when before were approximately 200,000 citizens) previously assigned to another Valencian Hospital, the *Hospital Doctor Peset* (finding F4). The uptake of this population was expected to initially cause an increase of 50 daily urgent admissions, progressively rising to 70. For this reason, three actions were taken, in which we can also find some of the findings previously detected by the methodology:

- A new surgical admissions unit was created to assess patients to be hospitalized.
- The number of beds assigned to home hospitalization was increased (see S6 Fig) to cover two more areas (Pediatrics and Neonatology) (previously chronic, mental health and pediatric oncology patients).

The pre-surgery planned-admission protocol was updated in early 2014. Whereas before this intervention, patients were admitted the night previous to surgery, they were now admitted on the morning of the intervention and they had a bed ready at midday after daily patient discharges. This meant an increase of the daily bed-occupation in the hospital and also on patient satisfaction, due to the shortening of the stay. The isolation of January 2014 in the multivariate analysis (see Fig 3) was probably caused by this change.

#### Discoveries and possible particular contributions

Time is a factor which has been studied as part of data quality dimension, generally leading to dimensions such as timeliness, currency, volatility, concordance or comparability [47–51]. Some of the data quality dimensions are used for validation of the quality of care [52]. The general contribution obtained by the TVA proposed is the use of the assessment of a data quality dimension in the monitoring of the interventions carried out by the hospital. For each intervention we want to highlight:

- I1. The relocation of the hospital. More than 1,800 professionals were involved in the operation and 40 ambulances were needed for the transfer. The data suffered a great impact, both multivariate and univariate (see Section *Findings*. and S1–S3 Figs) and the impact on the whole set of variables was monitored by the TVA proposed in the present study. The impact on the variables was produced not only in the expected ones. In this sense, the TVA monitoring may provide an added value when is used as a tool for “real-time” detection.
- I2. Services reconfiguration due to changes in hospital management policies by logistic relocations (see Section Services re-configuration). Some changes in services and treatment areas occurred during the study period. In addition to its capacity for management process control, our proposed methodology can reveal information and subsequent considerations to help in data reuse, for example for prediction purposes as well as for observational studies involving the comparison of different services during a period of reduced data quality.
- I3. Reconfiguration of care areas due to PR decisions (See Section Care services distribution). After a logistic relocation, the hospital activity probably suffered several unexpected difficulties. These difficulties led to PR decision-making that can be monitored by the proposed TVA which may be useful to create “PR effectiveness indicators” to be used as a background for future interventions.
- I4. The inclusion of 80,000 patients from another Valencian Hospital. It would have produced a hospital overcrowding if the interventions (detected by the proposed approach) had not been taken. The most important intervention produced an increase in the percentage of surgeries carried out on the day of admission, rising from 0% to 75%, avoiding a collapse due to an increase in the percentage of beds occupied, which rose to 97% from the previous 82%. It is worth mentioning that one of the challenges in the rise in the number of patients was the integration of computer data into the Business Intelligence used by the hospital. The knowledge of both the increased population assigned to the HFE and the pre-surgery planned-admission protocol change may influence the corresponding data for descriptive or research purposes.

### Limitations

One of the principal advantages of the TVA methodology used here is its capacity to analyze a great number of variables in a single iteration. This may also influence the loss of information about what is happening and where at a higher granularity, implying the need for knowledge in the field of study. For instance, finding 4 presented in Section *Results* firstly was considered as two findings, the hospital PR expertise was needed to understand the scope of the intervention associated with this finding.

Using a single-component PCA reduces the dimensionality of the iteration of the multivariate analysis and may smooth other discoveries with less impact in global terms, making the univariate iteration necessary not only to explain but also to detect. The use of other non-linear reduction methods such as t-distributed Stochastic Neighbor Embedding [53] or machine learning approaches [54,55] may have a better fit in certain cases and also contribute in the pursuit of interactions between variables.

Faulty healthcare processes are one of the main causes of practitioners making technical mistakes [56], can compromise patient safety and even cost lives [57]. However, in this study, we did not focus our attention on detecting processes for improvement, which is another possible application of the methodology for healthcare management.

### Future work

In line with the present study, and to overcome the limitation mentioned above, we aim to develop an automated algorithm that can suggest the origin of the multivariate changes in terms of a set of implicated variables or their interactions.

## Conclusions

Temporal variability in EHR may be considered as an intrinsic data quality feature due to its implications for data reuse. In this work, we have demonstrated how data changes over time and how the statistical distributions of EHR are biased by clinical and management PR interventions in the case of a Valencian hospital over seven years. Analyzing the temporal data variability by means of TVA has the potential not only to detect but also to monitor Big Data hospitalization resources, in order to improve the assessment of PR in healthcare systems.

### Acronyms for months

Jan: January, F: February, m: March, a: April, M: May, j: June, J: July, A: August, S: September, O: October, N: November, D: December.

## Supporting information

S1 Appendix Data base description. Consort diagram and description of categorical variables.
(DOCX)

S1 Fig Supplementary evidence for I1. PDF-SPC, IGT plot, its clustering by DBscan and Heat Map for the variable DischargeReason. An abrupt change is detected at the end of 2010 when the hospital relocation took place. The admittance of patients in delicate health states reduced the number of discharges under “Healing or improvement”.
(TIF)

S2 Fig Supplementary evidence for I1 and I2. The color density in February 2011 band shows the increase in the percentage due to the last month of the relocation. The lower number of observed hospitalizations implies a lower number of interventions and an increase in urgent admissions.
(TIF)

S3 Fig Supplementary evidence for I1 and I2. PDF-SPC of HospitalTransfer. The abrupt changes detected in late 2010 and early 2011 are the results of the hospital relocation.
(TIF)

S4 Fig Supplementary evidence for I2. IGT Plot and Temporal Heat Maps for the Service configurations.
(TIF)

S5 Fig Supplementary evidence for I3. PDF-SPC for the HospitalTransfer variable. The change caused by a) the opening of the chronic patient’s area in the old facilities in early 2013, and b) the new readmittance to the new facilities in early 2016. The changes were detected after removing the cases prior to March 2011 –with the purpose of avoiding the loss of change detection due to the high impact of the hospital relocation-.
(TIF)

S6 Fig Supplementary evidence for I4. Temporal absolute count for the discharge destination variable. Since the opening of the chronic service in the old hospital facilities, a new code–in which patients who would be treated in the chronic area were included- was created “Outpatient care”. This implies a decrease in the number of patients who were sent home until 2016 when the chronic area was closed.
(TIF)

S7 Fig Supplementary evidence for I4. Temporal relative count for the variable which records the length–in days- of the stay for each hospitalization. This image shows that the percentage of 1-day stays increases in detriment of 2-days stays. This shows that the aim of reducing the length of stay, as described in M4, was successful.
(TIF)
