# Supplementary material for: Temporal variability analysis reveals biases in electronic health records due to hospital process reengineering interventions over seven years
Source: PLoS One. 2019 Aug 7;14(8):e0220369. doi: 10.1371/journal.pone.0220369 (PMC6685618; doi:10.1371/journal.pone.0220369)
Supplement: S1 Appendix — Consort diagram and description of categorical variables. (DOCX) [file pone.0220369.s001.docx]

Temporal variability analysis reveals biases in electronic health records due to hospital process reengineering interventions over seven years

**S1 Appendix: Data base description**

Francisco Javier Pérez-Benito^a^, Carlos Sáez^a^, J. Alberto Conejero^b*^, Salvador Tortajada^a,c,d^, Bernardo Valdivieso^c^, Juan M. García Gómez^a,c,d^

^a^ Biomedical Data Science Lab, Grupo de Informática Biomédica, Instituto de Aplicaciones de las Tecnologías de la Información y de las Comunicaciones Avanzadas, Univeritat Politécnica de València, València, Spain.

^b^ Instituto Universitario de Matemática Pura y Aplicada, Universitat Politécnica de València, València Spain.

^c^ Unidad conjunta de investigación en reingeniería de procesos socio-sanitarios. Instituto de Investigación Sanitaria La Fe. Hospital Universitario La Fe, València, Spain.

^d^ Red de Investigación en Servicios de Salud en Enfermedades Crónicas (REDISSEC), València, Spain.

** Corresponding Author*

*E-mail addresses:* [*aconejero@upv.es*](mailto:aconejero@upv.es) *(JAC)*

This document presents the consort diagram for the data base considered for this study, the tables needed for the comprehension of the multi-category variables –understanding multi-category variables with more than 3 options-.

Data base description

1.
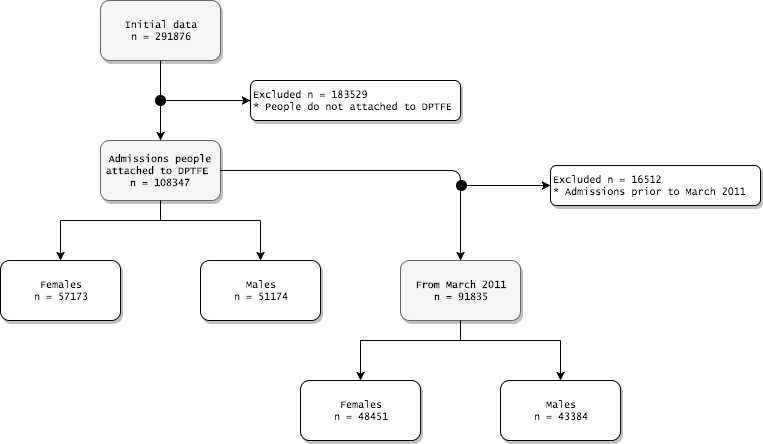
Consort Diagram

Figure A1: Consolidated Standard Reporting Trials (CONSORT) flow diagram of the case study of the HFE hospitalization data base.

1. Dictionaries

**Service codes.** Used for the variables *IncomeServiceCode*, *RealServiceCode* and *DischargeServiceCode*:

| **Code** | **Description** | **Code** | **Description** | **Code** | **Description** |
| --- | --- | --- | --- | --- | --- |
| HUDO | Pain unit | HURO | Urology | HREI | Child rheumatology |
| HPSA | Adolescent psychiatry | HHEM | Hematology & hemotherapy | HCGI | Pediatrics surgery |
| HREU | Rheumatology | HCCV | Cardiovascular surgery | HUTA | Eating disorders unit |
| HORP | Orthoptics & pleoptica | HTRA | Traumatology | HRTE | Radiation oncology |
| HDII | Child Digestive | HNAI | Child pneumology & allergies | HCMX | Maxillofacial surgery |
| HMUR | Short stay unit | HCG2 | General surgery & digestive system II | HSEP | Septic unit |
| HECI | Child endocrinology | HNEN | Neonatology | HCDG | Digestive surgery |
| HNCI | Child neurosurgery | HCUR | Curietherapy | HCPI | Child plastic surgery |
| HCVI | Child cardiovascular surgery | HUHP | Hepatobiliopancreatic | HPED | General pediatrics |
| HRXD | Radiology | HMIN | Internal medicine | HALI | Child allergy |
| HRQU | Burn care resuscitation | HOFT | Ophtalmology | HCAR | Cardiology |
| HGIN | Gynecology | HONI | Child oncology | HQUE | Burn care |
| HUEG | Esophagogastric surgery | HCTO | Thoracic surgery | HSII | Child psychiatry |
| HREP | Reproduction | HCIR | General surgery & digestive system | HMNU | Nuclear medicine |
| HRHB | Rehabilitation | HUHT | Hemostasis & thrombosis | HCAI | Child cardiology |
| HECR | Endogrinology & nutrition | HNCG | Neurosurgery | HCOT | Orthopedic & traumatology |
| HEPR | Refractory epilepsy | HOFI | Child ophtalmolgy | HMDH | Hepatology |
| HUCP | Pediatric ICU | HRER | Anesthesia-resuscitation (RET) | HUEI | Infectious diseases unit |
| HORL | Otolaryngology | HUEM | Metabolic endocrine surgery | HUMI | Intensive medicine |
| HPSI | Psyquiatry | HOBS | Obstetrics | HCLP | Coloproctology surgery |
| HUMM | Functional Breast Cancer Unit | HNER | Neurology | HALE | Allergy |
| HPIN | Pediatric infectious | HURQ | Raquis unit | HHMI | Child hematology |
| HCVA | Angiology & vascular surgery | HDER | Dermatology | HNMI | Child pneumology |
| HURI | Child urology | HOTI | Child orthopedic & traumatology | HCMI | Child maxillofacial surgery |
| HMDG | Gastroenterology | HCLP | Plastic surgery | HCEP | Short stay unit & wall |
| HORI | Child otolaryngology | HUML | Medium-long stay unit | HNEM | Pneumology |
| HONC | Oncology | HNRI | Neuropediatrics | HULM | Spinal cord injury unit |
| HMIF | Lower limb unit | HREM | Anesthesia-resuscitation (MAT) | HUTP | Lung transplantation unit |
| HMET | Child metabolic diseases | HNEF | Child rheumatology | HLIT | Lithotripsy |
| HNFI | Child nephrology | HREA | Resuscitation | UCSI | Non-admittance surgical unit |

Table 1: List of variables contained in the study. Service identifier

**Hospital codes.** Used for variable *HospitalTransfer*:

| **Code** | **Description** | **Code** | **Description** | **Code** | **Description** |
| --- | --- | --- | --- | --- | --- |
| 1000 | Inst. Oftalmologico Valencia | 1101 | Hospital de Vinaros | 1102 | Hospital Gral. de Castellón |
| 1103 | Hospital de la Magdalena | 1104 | Hospital de la Plana | 1111 | Hospital 9 de Octubre |
| 1150 | Hospital Prov. de Castellón | 1191 | Termalismo Heliomar Benicassim | 1192 | Mutua de Azulejeros, Onda |
| 1369 | Hospital Intern. Medimar | 1537 | Hospital Rey Don Jaime | 1717 | Hospital S. Jaime Torrevieja |
| 1901 | Clínica Santa Teresa | 1902 | Ntra. Sra. de la Misericordia | 1903 | Ctro de Rehabilitación, Onda |
| 2021 | Hospital de Levante | 2101 | Hospital de Sagunto | 2102 | Hospital Arnau Vilanova |
| 2103 | Hospital Doctor Moliner | 2104 | Hospital de Requena | 2105 | Hospital La Fe (Campanar) |
| 2106 | Hospital Dr Peset I Aleixandre | 2107 | Hospital Clínico Universitario | 2108 | Hospital Malvarrosa |
| 2109 | Hospital Francesc de Borja | 2110 | Hospital Lluis Alcanys Xativa | 2111 | Hospital d’Ontinyent |
| 2115 | Hospital Rehabilitación La Fe | 2125 | Hospital Maternal La Fe | 2135 | Hospital Infantil La Fe |
| 2145 | Hospital La Fe. Esc Enfermería | 2150 | Hospital General de Valencia | 2155 | La Fe Bulevar |
| 2169 | Vissum Inst. Oftalmológico | 2191 | Centro Rehabilitación Levante | 2192 | Inst. Valenciano Oncología |
| 2194 | Hospital Santa Lucía | 2526 | Hospital de Torrevieja | 2837 | Hospital de Manises |
| 2840 | Hospital de Denia | 2844 | Hospital la Pedrera | 2901 | Clínica Blanquer |
| 2902 | Cl. Sagrada Familia | 2903 | Casa de Reposo San Onofre | 2904 | Clínica Virgen del Consuelo |
| 2905 | Hospital de Valencia al mar | 2906 | Clinica Quirón de Valencia | 2907 | Clínica Casa de la Salud |
| 2908 | Hospital de Mislata (Militar) | 2909 | Hospital Santa Lucía | 2910 | Hospital Padre Jofre |
| 2993 | FISABIO | 3050 | Hospital del Vinalopo | 3082 | Hospital de Lliria |
| 3101 | Hospital Marina Alta | 3102 | Hospital Vila-Joiosa | 3103 | Hospital Verge del Lliris |
| 3104 | Hospital de Elda | 3105 | Hospital General de Alicante | 3106 | Hospital S. Vicent del Raspeig |
| 3107 | Hospital D’Elx | 3108 | Hospital de Orihuela | 3110 | Hospital Sant Joan. Alicante |
| 3116 | Fundacion Lluis Alcanyis | 3150 | Hospital Provincial de Alicante | 3191 | S.S. FCO. de Borja. Fontiles |
| 3192 | Clinica Vistahermosa | 3193 | Clínica Velazquez II. Alicante | 3194 | S. Perpetuo Socorro. Alicante |
| 3901 | Centro Médico San Carlos | 3902 | Sanatorio San Jorge | 3903 | Clínica Benidorm |
| 3904 | Instituto Geriátrico de Levant | 3905 | Policlínico S. Carlos de Denia | 3906 | Clínica Oftalmológica Buigues |
| 3907 | Cl. Médico Quirúrgica C. Jardín | 3908 | Cl. Villamartin de Orihuela | 3909 | Psiq. Penitenciario Fontcalent |
| 3910 | Hospital PSIQ. Provincial Alicant | 3911 | Levante Mediterranea Matepss | 7777 | Hospital de la Ribera |
| 7778 | Hospital 9 de Octubre | 7779 | Other Country | 8888 | F.Oftalmológica Mediterránea |
| 9040 | Hospital La Fe Unificado | 9998 | Barraca de Aguas Vivas | 9999 | Other Community |

Table 2: List of hospitals contained in the HFE registries.

**Admission reasons.** This variable shows the reason for hospitalization:

| **Code** | **Description** |
| --- | --- |
| 0 | Undetermined |
| 1 | Examination |
| 2 | Common disease |
| 4 | Accident at work |
| 5 | Casual accident |
| 6 | Self-injury |
| 7 | Aggression |
| 8 | Birth |
| 9 | Others |
| 10 | Pathological new-born |
| 11 | Urgent outpatient complication |
| 12 | Surgery complication |
| 13 | Day hospital complication |
| 14 | Interventionism complication |
| 15 | Infract |
| 19 | Urgent outpatient complication |
| 20 | Patent from other hospital planned |
| 60 | An influenza research case |
| 61 | An influenza probable case |
| 62 | An influenza confirmed case |
| 63 | A influenza dismissed case |
| 90 | UCSI episode complication |
| 99 | Disaster |

Table 3: List of hospitalization reasons cointained in the study.

**Discharge reasons.** The information collected in this variable indicates the reasons for discharge:

| **Code** | **Description** |
| --- | --- |
| 1 | Healing or improvement |
| 2 | Voluntary discharge request |
| 3 | Transfer |
| 4 | Exitus |
| 5 | Other |
| 6 | In extremis |

Table 4: List of discharge reasons contained in the study.

**Destination after discharge.** This variable contains the patient follow-up method after discharge:

| **Code** | **Description** |
| --- | --- |
| 1 | Day hospital |
| 2 | Discharge home |
| 3 | Outpatient care |
| 5 | Specialty center |
| 6 | Emergency department |
| 8 | Escaped |
| 9 | Others |
| 10 | Medium and long stay hospitals |
| 11 | Nursing home or socio-health center |
| 12 | General practitioner |
| 13 | Disciplinary discharge |

Table 5: List of discharge destinations contained in the study.
