# Supplementary figures and images for: Temporal variability analysis reveals biases in electronic health records due to hospital process reengineering interventions over seven years
Source: PLoS One. 2019 Aug 7;14(8):e0220369. doi: 10.1371/journal.pone.0220369 (PMC6685618; doi:10.1371/journal.pone.0220369)

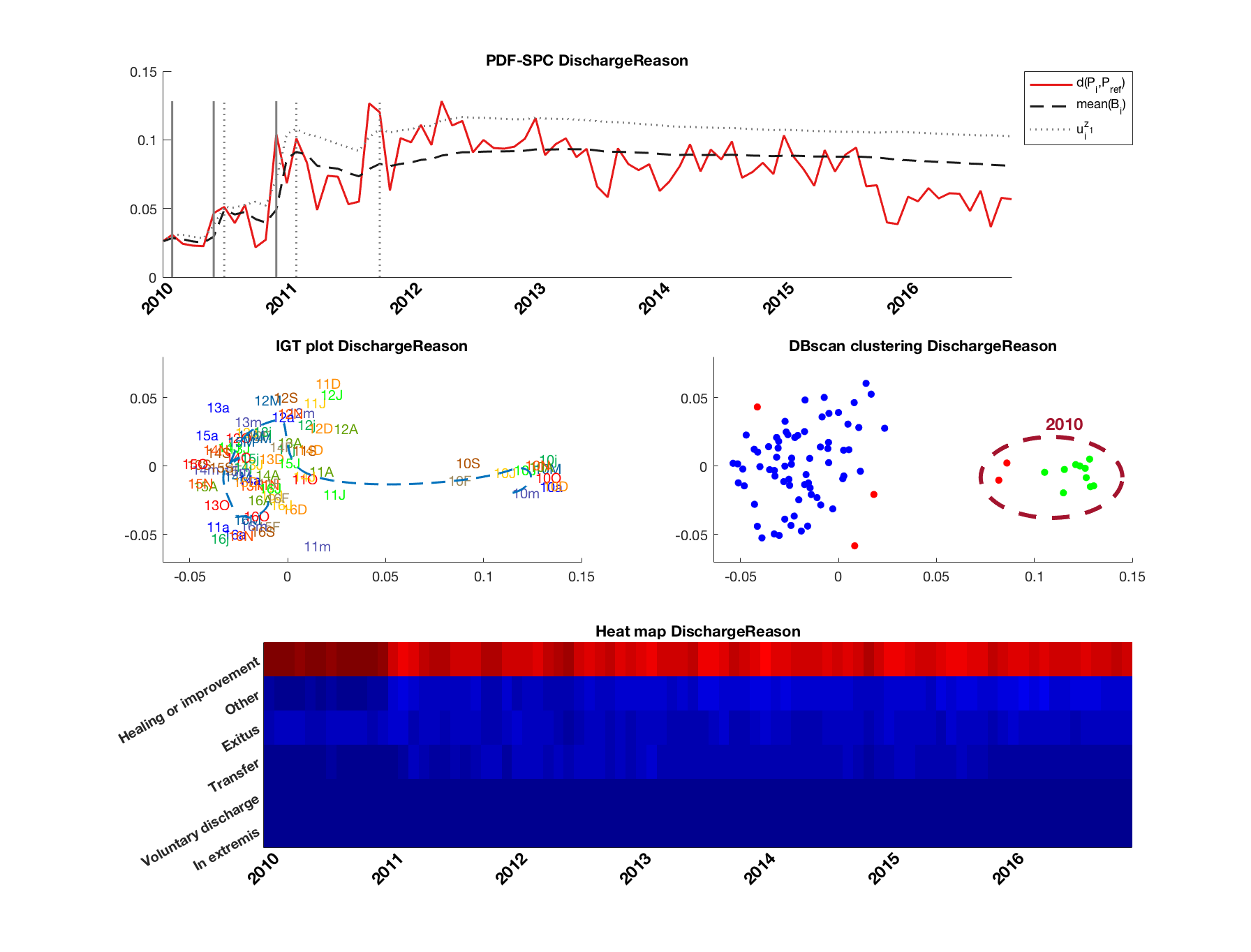

Supplement: S1 Fig — PDF-SPC, IGT plot, its clustering by DBscan and Heat Map for the variable DischargeReason. An abrupt change is detected at the end of 2010 when the hospital relocation took place. The admittance of patients in delicate health states reduced the number of discharges under “Healing or improvement”. (TIF) [file pone.0220369.s002.tif]

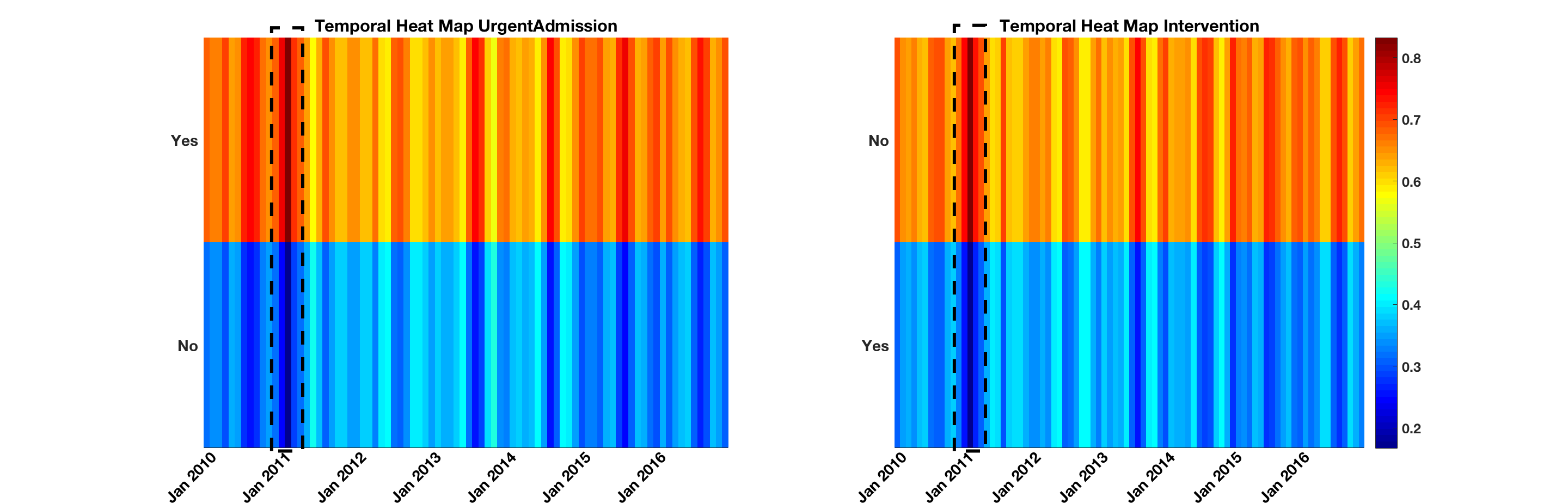

Supplement: S2 Fig — The color density in February 2011 band shows the increase in the percentage due to the last month of the relocation. The lower number of observed hospitalizations implies a lower number of interventions and an increase in urgent admissions. (TIF) [file pone.0220369.s003.tif]

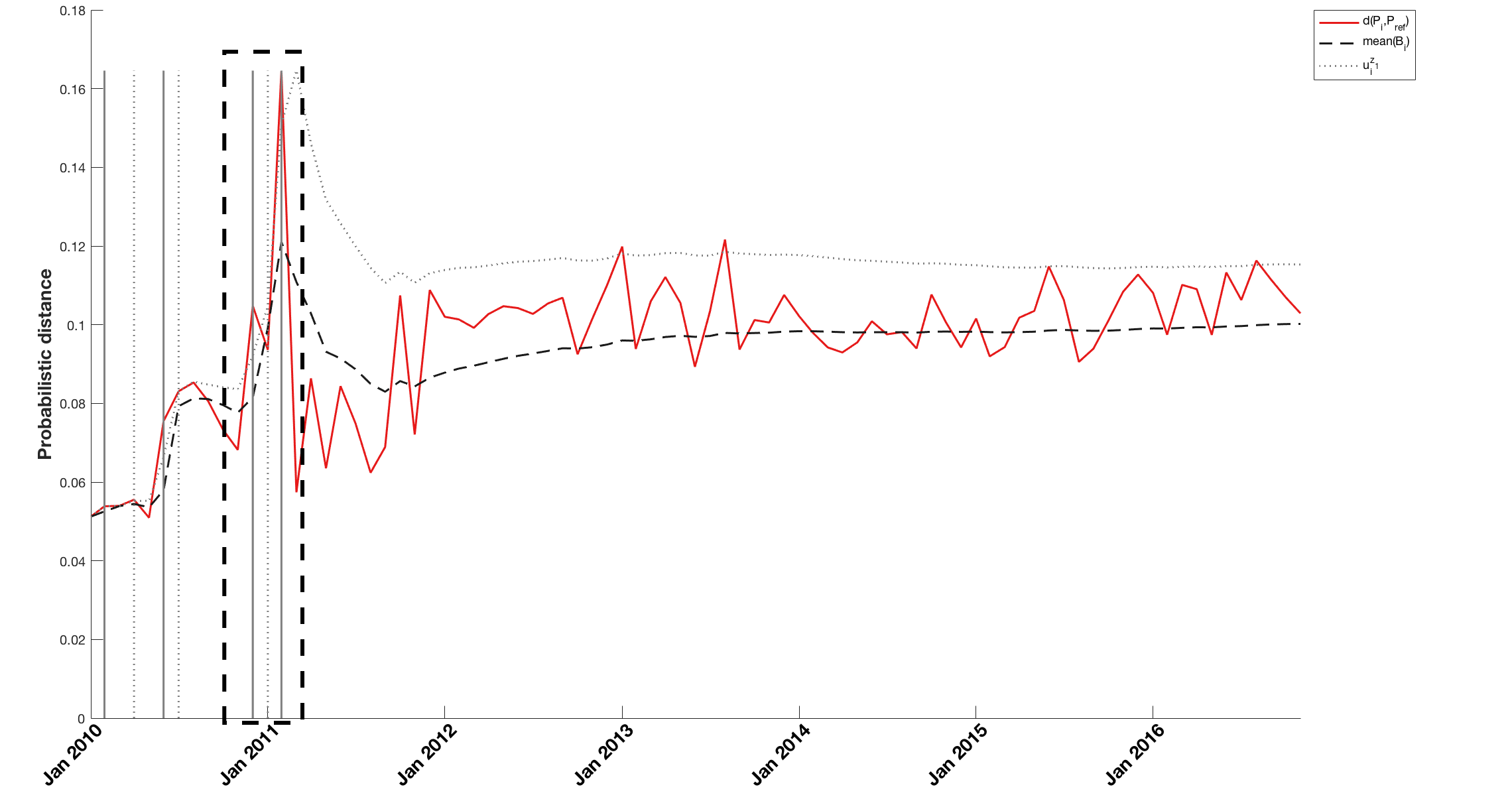

Supplement: S3 Fig — PDF-SPC of HospitalTransfer. The abrupt changes detected in late 2010 and early 2011 are the results of the hospital relocation. (TIF) [file pone.0220369.s004.tif]

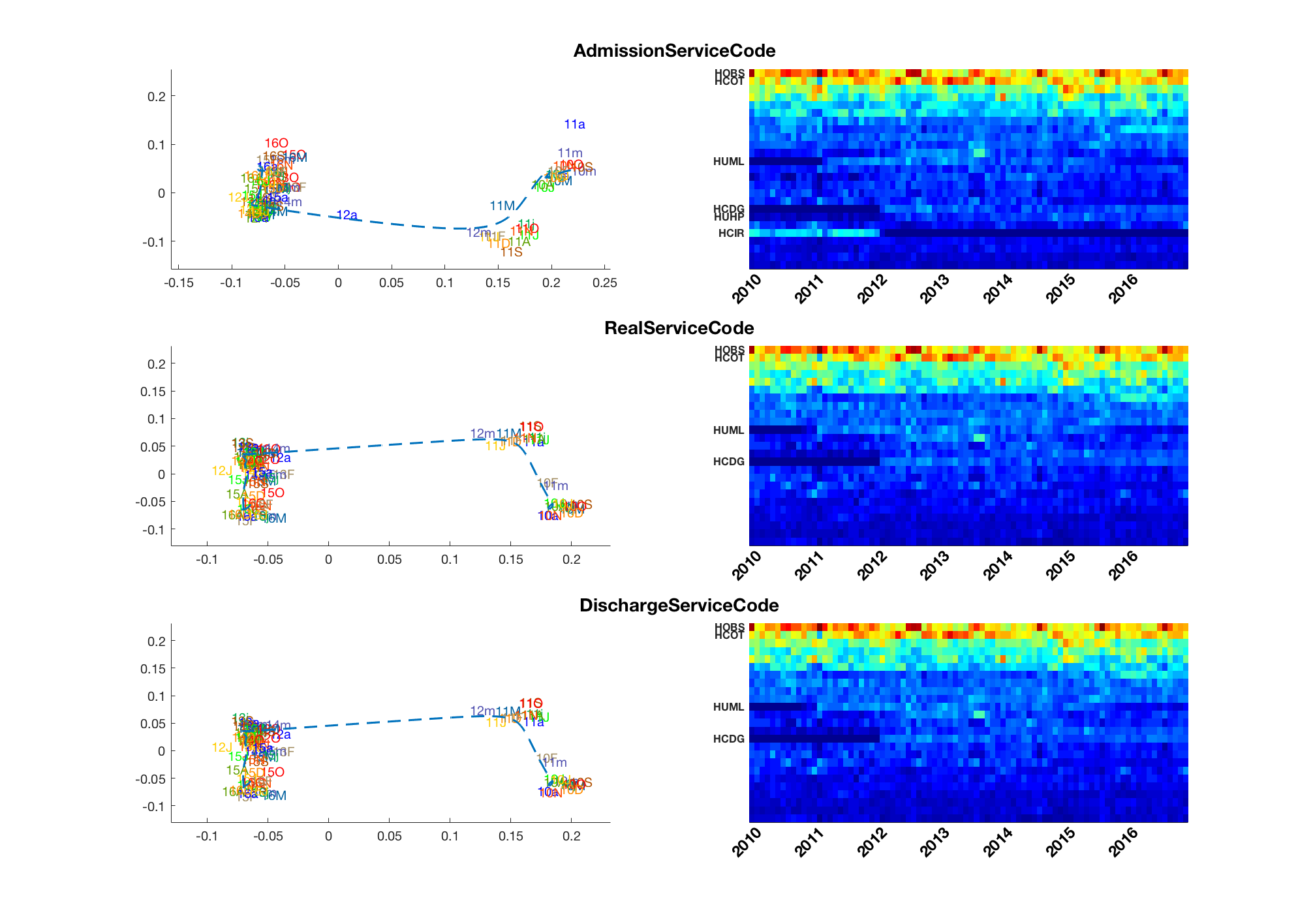

Supplement: S4 Fig — IGT Plot and Temporal Heat Maps for the Service configurations. (TIF) [file pone.0220369.s005.tif]

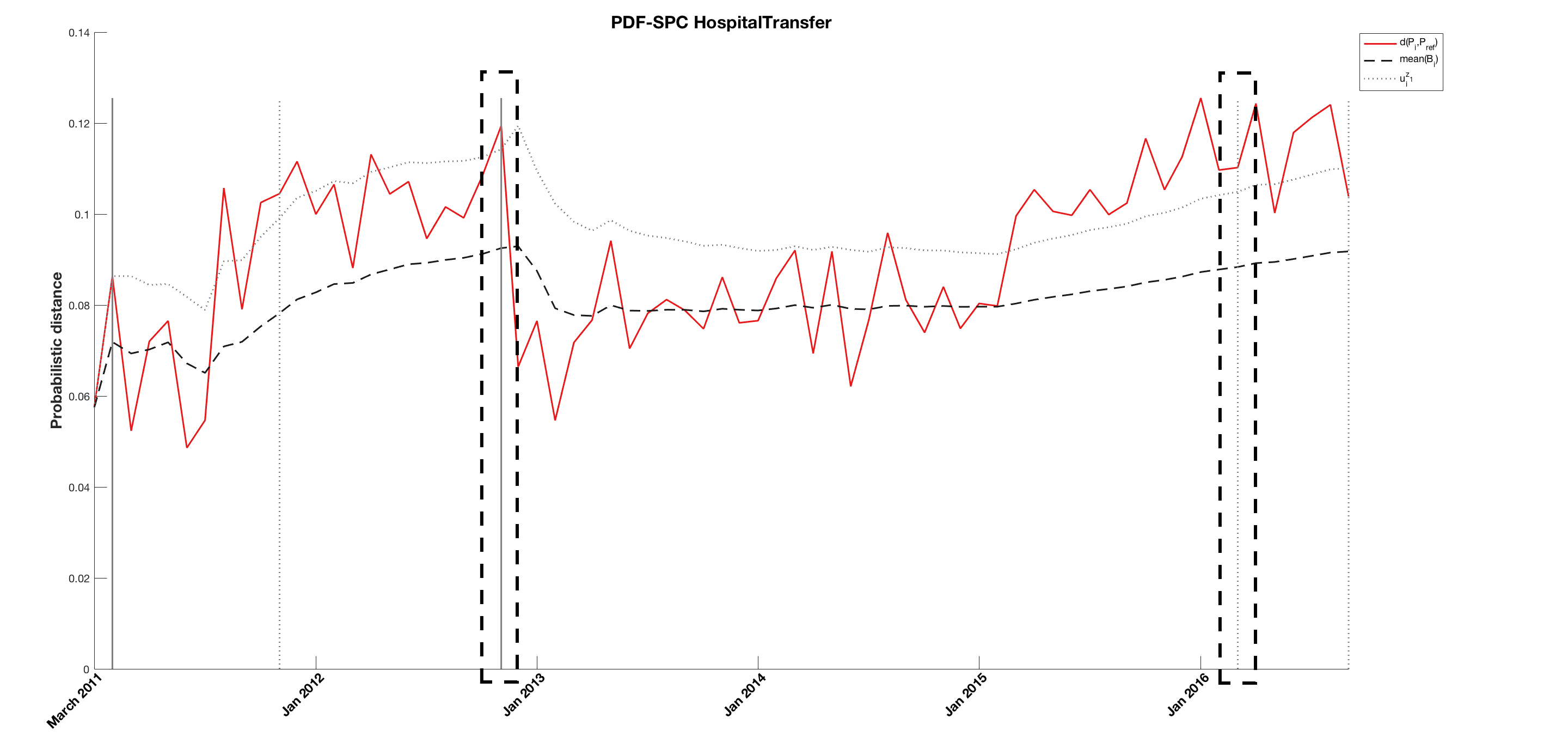

Supplement: S5 Fig — PDF-SPC for the HospitalTransfer variable. The change caused by a) the opening of the chronic patient’s area in the old facilities in early 2013, and b) the new readmittance to the new facilities in early 2016. The changes were detected after removing the cases prior to March 2011 –with the purpose of avoiding the loss of change detection due to the high impact of the hospital relocation-. (TIF) [file pone.0220369.s006.tif]

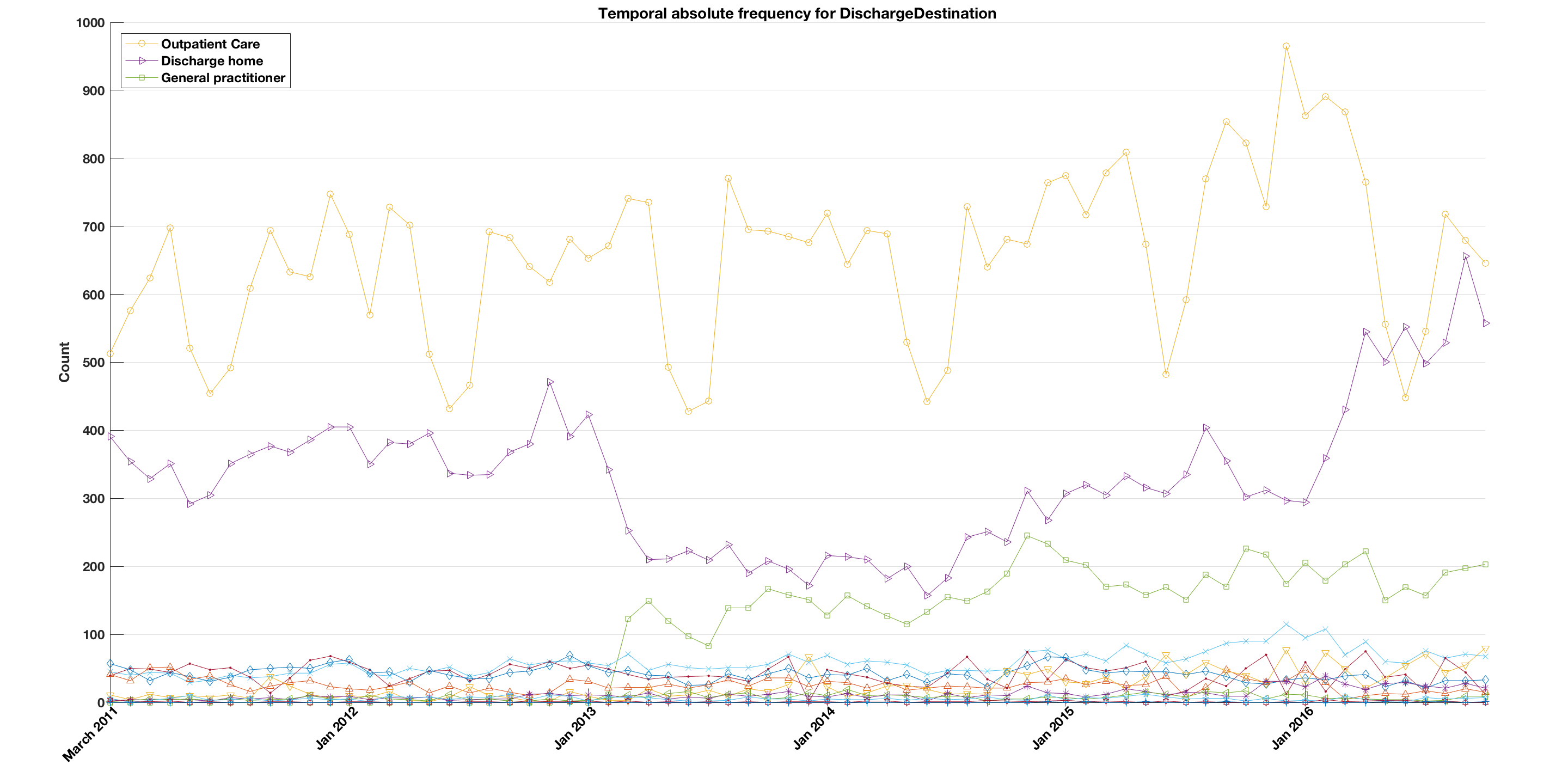

Supplement: S6 Fig — Temporal absolute count for the discharge destination variable. Since the opening of the chronic service in the old hospital facilities, a new code–in which patients who would be treated in the chronic area were included- was created “Outpatient care”. This implies a decrease in the number of patients who were sent home until 2016 when the chronic area was closed. (TIF) [file pone.0220369.s007.tif]

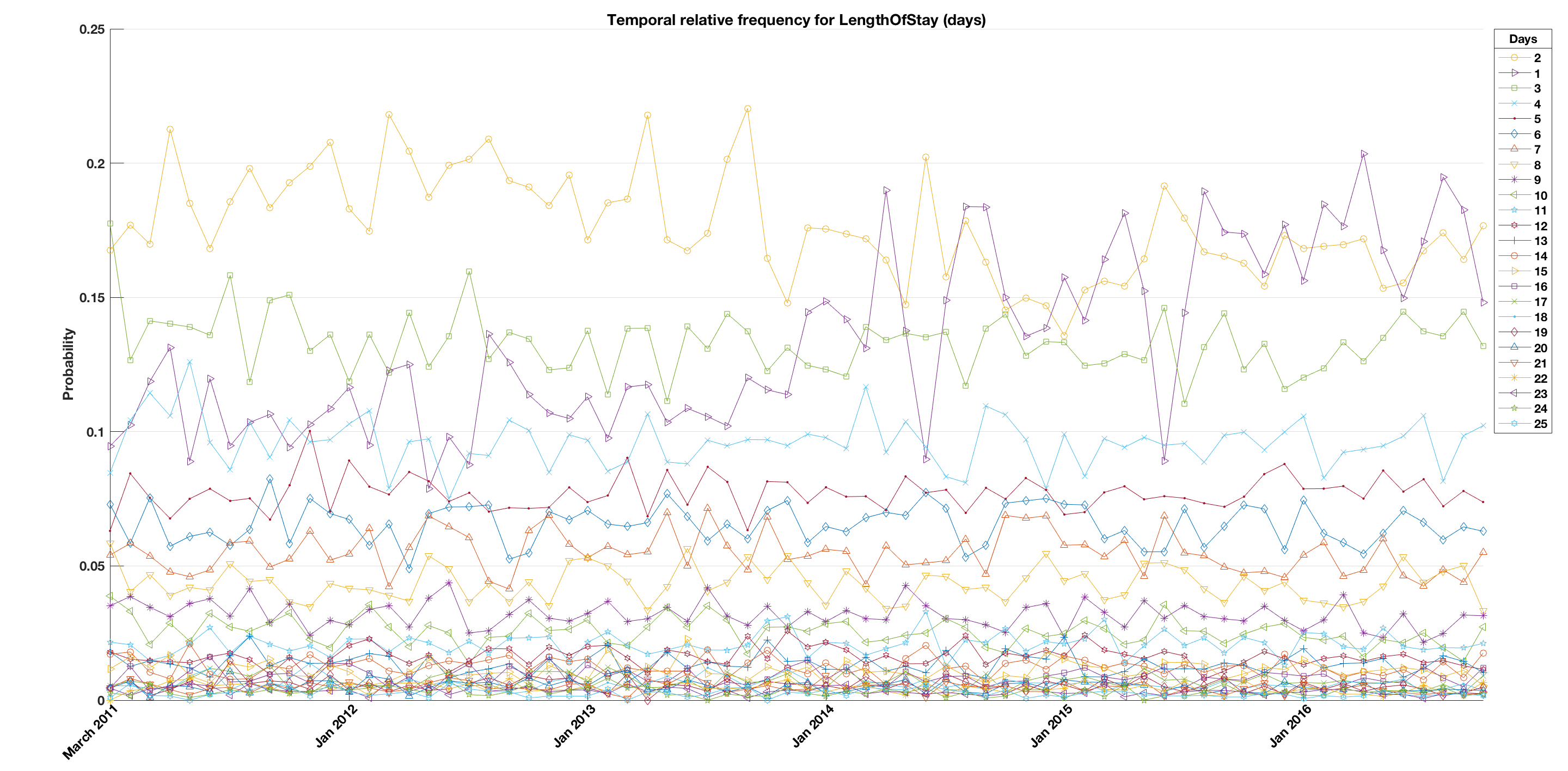

Supplement: S7 Fig — Temporal relative count for the variable which records the length–in days- of the stay for each hospitalization. This image shows that the percentage of 1-day stays increases in detriment of 2-days stays. This shows that the aim of reducing the length of stay, as described in M4, was successful. (TIF) [file pone.0220369.s008.tif]
